# How Epstein–Barr Virus and Kaposi’s Sarcoma-Associated Herpesvirus Are Maintained Together to Transform the Same B-Cell

**DOI:** 10.3390/v13081478

**Published:** 2021-07-28

**Authors:** Arthur U. Sugden, Mitch Hayes, Bill Sugden

**Affiliations:** McArdle Laboratory for Cancer Research, University of Wisconsin-Madison, Madison, WI 53705, USA; ausugden@wisc.edu (A.U.S.); mhayes1@oncology.wisc.edu (M.H.)

**Keywords:** KSHV, EBV, model, dual-infection

## Abstract

Epstein–Barr virus (EBV) and Kaposi’s sarcoma-associated herpesvirus (KSHV) independently cause human cancers, and both are maintained as plasmids in tumor cells. They differ, however, in their mechanisms of segregation; EBV partitions its genomes quasi-faithfully, while KSHV often clusters its genomes and partitions them randomly. Both viruses can infect the same B-cell to transform it in vitro and to cause primary effusion lymphomas (PELs) in vivo. We have developed simulations based on our measurements of these replicons in B-cells transformed in vitro to elucidate the synthesis and partitioning of these two viral genomes when in the same cell. These simulations successfully capture the biology of EBV and KSHV in PELs. They have revealed that EBV and KSHV replicate and partition independently, that they both contribute selective advantages to their host cell, and that KSHV pays a penalty to cluster its genomes.

## 1. Introduction

Epstein–Barr Virus (EBV) and Kaposi’s sarcoma-associated herpesvirus (KSHV) are two human γ-herpesviruses; both are maintained extra-chromosomally in proliferating cells and both cause several kinds of cancers in people. However, each has evolved to use markedly different mechanisms to partition its DNAs to daughter cells. EBV segregates them quasi-faithfully [1] and KSHV often clusters and segregates them randomly [2]. Both viruses can infect the same primary B-cells in which they induce primary effusion lymphomas (PELs) [3,4,5].

Many of the steps in the replication of EBV genomes in latently infected cells have been characterized in bulk populations and by live cell imaging [1,6,7,8]. They then have been computationally modeled to reveal new insights into these steps [1]. Similarly, how KSHV replicates its DNA has been experimentally measured and subsequently simulated to identify unknown facets of its replication [2,9,10,11,12,13,14,15]. It has become possible to infect primary B-cells with both viruses to establish stably infected, transformed populations, allowing us now to examine their replication computationally together in the same cells [16]. How these disparate viral replicons maintain themselves in the same cells is a fascinating puzzle we address here.

We have two sets of information to help us with this puzzle. First, we have developed a means to infect primary human B-cells successfully with both EBV and KSHV [16]. Exposing these cells to both viruses optimally yielded 1 clone per 10,000 cells that was stably transformed and maintained each virus [16]. Both viral replicons were eventually maintained in these growing cells at 10–30 copies per cell on average.

The second set of information for this puzzle comes from our measurements of the synthesis and partitioning of each of these replicons individually in live, proliferating cells and our developing simulations that model these events accurately [1,2]. We found that 84% of EBV replicons are synthesized each cell cycle and 88% of these replicated molecules are segregated symmetrically to daughter cells by associating with sister chromatids [1]. Moreover, 90–92% of KSHV plasmids are synthesized each cell cycle, but in striking contrast to EBV, they can aggregate into clusters, which are then partitioned randomly. For example, these viral replicons often form clusters of tens of molecules that can be inherited by single daughter cells [2].

We have now developed computer simulations that describe how these mechanistically distinct viral replicons are maintained successfully in the same proliferating, that is, transformed, cells. Our previous simulations of EBV and KSHV replicons each in different cells did not require any consideration of the potential selective advantages the replicons afford their hosts [1,2]. Modeling how these two genomes are maintained together in the same cells has required an additional consideration of the mechanisms and consequences of positive selection. These new simulations delineate both the positive and negative selective pressures that are necessary to allow both replicons to remain at equilibrium in the dually infected, transformed cells as characterized in cell culture [16].

Simulating the replication and partitioning of EBV and KSHV together in the same cells has yielded three insights into the properties of these viral replicons and the functions they provide their host cells. We have found that EBV and KSHV replicate and partition as independent replicons in the same cell, that both viruses contribute selective advantages to their host cell, and that KSHV pays a penalty by clustering its genomes.

## 2. Materials and Methods

We built upon previous models [1,2] to develop this simulation framework. It can accept parameters representing latent infections by herpesviruses, including positive and negative selective advantages, as well as identify the most realistic parameters with which to model dually infected cells. The maintenance of plasmids was modelled as the combination of replication, in which a plasmid may be duplicated in S phase, and partitioning, when the daughter plasmids are distributed to daughter cells. Because KSHV plasmids can form clusters [2], we incorporated the ability of individual plasmids to come together to form clusters and for clusters to break up, resulting in smaller clusters or individual plasmids. The program created to use this model, LatentPlasmidPopulation, is written for Python 3.7+. The source code and extensive documentation can be found at the Github repository found at https://github.com/asugden/plasmid_rep (accessed on 7 July 2021).

We used a combination of in vitro and in silico experiments and found six parameters to describe the complexities of herpesvirus replication (some of which were found in previous work to be dispensable for EBV and KSHV, but have been retained in the simulation for other replicons). Whether the parameters were determined by experiments in vitro, in silico, or simply set is defined in Table 1 and are further elaborted in Figure S1. S-phase duplication probability defines the per-plasmid duplication rate (0.84 for EBV, 0.98 for KSHV). Plasmid repulsion–attraction defines an axis of plasmids or clusters of plasmids binding to sister chromatids and segregating equally at a value of 0 to fully forming clusters and segregating randomly at a value of 1 (0.12 for EBV and 1.0 for KSHV). Positive selection, which is a term for a selective advantage, applies to both EBV and KSHV and is represented as a sigmoid scaled to pass through 0 and asymptotically approach 1 (optimized to be 0.10 for EBV and 0.20 for KSHV). Three additional parameters are specific to KSHV: the probability of cluster breakup, the CRP (a subset of Dirichlet processes termed the Chinese restaurant process) parameter, and a coefficient for negative selection for large-size clusters. The probability of cluster breakup defines the probability that a cluster will enter the CRP process after duplication (0.80 for KHSV). The CRP defines the size and number of the resulting clusters after breakup (shown in Figure 3C,D, optimized to be 0.50). The coefficient of negative selection as a function of cluster size is modeled as an exponential decay scaled by the duplication probability (optimized to be 0.07). 

Six parameters allow simulation of the differing conditions of dual infections in vitro. The initial population is set by the number of cells to simulate, which determines the number of simulated cells with at least one plasmid (set to 5000 for these simulations). When cells have zero plasmids, they are modeled separately for the sake of speed. This choice improves the accuracy of models with most cells containing no plasmids. The initial distribution can be set using a normal distribution by defining the mean, µ, and standard deviation, σ, or by modeling it via the multiplicity of infection (m.o.i.), modeled as a Poisson distribution and set by the variable λ (measured to be 2 for EBV and 0.02 for KSHV). The population is then simulated, first for a number of “burn-in generations” to get the population to equilibrium (set to 0 except in the case of identifying the best-fit value for negative selection in KSHV that produces a mean plasmid replication rate of 0.92, in which it was set to 30), and finally simulated for a number of generations (set to 50 for the figures shown here).

To identify the optimal value for negative selection, we extended the model to track and average the replication probability across all plasmids within all clusters. We varied the negative selection parameter and base duplication rate to identify pairs of parameters that produced a replication probability of 0.90–0.92. Finally, we simulated distributions of KSHV with each of those pairs of parameters, settling on 0.07 and 0.98, respectively, to best reproduce the populations identified in [16]. The methods for establishing the computational parameters used in these simulations are given in Supplementary Table S1. 

**Figure 1:**
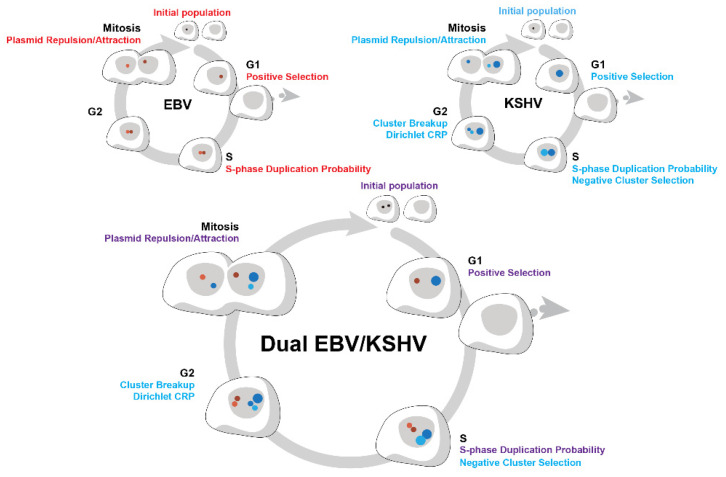
The different computational steps in developing simulations of the replication and partitioning of Epstein–Barr virus (EBV) in cells (upper left), of Kaposi’s sarcoma-associated herpesvirus (KSHV) in cells (upper right), and of both replicons in the same cells (below) are depicted. The parameters used at the different stages of the cell cycle are shown and defined in the Materials and Methods. Whether they have been determined by experiments in vitro, in silico, or set as a simulation parameter is detailed in Table 1. The red dots represent EBV replicons, the blue dots represent KSHV replicons, and their sizes reflect single or clustered molecules. The shades of these colors identify the parental cell from which each replicon is segregated. Cells that lose all viral genomes leave the cell cycle to die.

The different computational steps in developing simulations of the replication and partitioning of EBV in cells, of KSHV in cells, and of both replicons in the same cells are depicted in Figure 1. The steps in simulating both replicons in the same cells are illustrated more extensively with their accompanying equations in Figure S1.

## 3. Results

### 3.1. EBV

**Figure 2:**
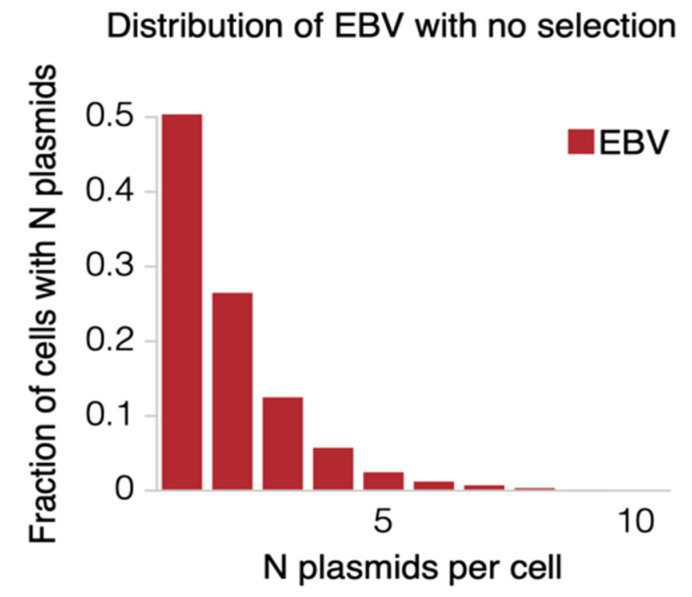
The distribution of the fraction of numbers of plasmids per cell in EBV is shown. This distribution was calculated from the findings of Nanbo et al. [1] following infection of primary B-cells with EBV at a multiplicity of infection of 2 after 50 generations of growth in culture. Not shown is the fraction of cells with zero plasmids per cell that die.

We want to model the replication and partitioning of both EBV and KSHV on infecting the same cell. We hypothesized that both replicons behave independently, so we first used our simulations of each alone in cells. Our previous simulations of EBV’s replication predicted that, over time, the number of molecules per cell declines to zero in the absence of selection [1]. This loss is a result of the rate of DNA synthesis of the viral genome being 84% per cell cycle and our simulation of it including no terms for any selective advantages provided by this genome. This rate of 84% of EBV genomes being synthesized per S-phase comes from a direct measurement and is robust. It was made by observing the synthesis of plasmids with OriP in live cells and validated by following the loss of intact EBV genomes from proliferating cells in the absence of selection [1]. It was independently confirmed using FISH to detect intact EBV genomes in four subclones of a lymphoblastoid cell line and using these measurements in a simulation that coupled the rate of genome synthesis with their partitioning to daughter cells [1]. The simulation accurately predicted the distribution of viral genomes in the parental population of cells [1]. Our previous work demonstrated that EBV’s replication could be accurately modeled with only two parameters: a probability of individual plasmid duplication and a probability of equal partitioning. The only remaining parameters described the initial conditions; in the case of dually infected cells, the EBV titers used were at a multiplicity of infection (m.o.i.) of approximately 2, which is the initial condition used in our current simulations. After 50 generations, these parameters yielded a distribution of the number of plasmids per cell that approximates an exponential decay (Figure 2). In this and all figures, the number of cells in a distribution with no plasmids is not shown. Such cells are lost as the cells are propagated in cell culture [16].

**Figure 3:**
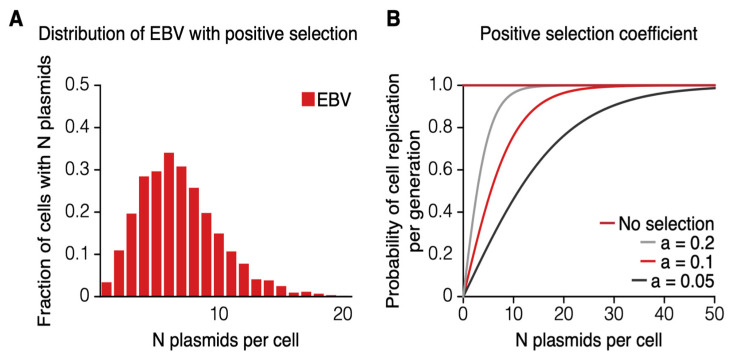
The distribution of the fraction of cells with given numbers of EBV plasmids per cell under positive selection is shown. (**A**) A distribution of the same form as in Figure 2 is depicted, but now calculated with a term for a positive selection. This positive selection is represented as an offset sigmoid function as follows: $F\left(x\right)=2\left(\frac{e^{ax}}{e^{ax+1}}\right)-1$, where x equals the number of EBV plasmids per cell and “*a*” is the coefficient for positive selective as shown in Figure 3B. In the depicted distribution, “*a*” was set optimally to 0.1, so that the distribution mirrors that found in cells after 100 generations of growth in culture [16]. (**B**) The contribution to the number of plasmids per cell derived by altering computationally the coefficient of positive selection in the offset sigmoid function. The probability of a cell replicating each generation on the Y-axis is increased as a function of the number of plasmids per cell on the X-axis such that, as the coefficient decreases, the effect of positive selection more strongly selects for higher numbers of plasmids per cell.

However, cells dually infected with both EBV and KSHV arrive at an equilibrium in which the average number of viral genomes for each virus ranges from 10 to 30 per cell without having radically higher numbers of EBV plasmids per cell [16]. We considered that one simple explanation to maintain such a distribution in equilibrium would be a positive selective advantage provided by EBV to these dually infected cells. EBV can confer multiple advantages on a cell including fostering its survival and its proliferation [17,18,19,20]. Forcing the loss of EBV from Burkitt lymphomas, for example, leads to their death [21]. We tested an alternate hypothesis that EBV would be replicated 100% of the time and found it did not yield a stable equilibrium. Rather, it yielded progressively higher copy numbers of plasmids per cell (Figure S2). We do not know what advantages EBV explicitly confers on dually infected cells, so we added a third parameter to our simulations that, as a single variable, can represent a wide range of possible selective advantages (Figure 1). The variable encompasses the probability with which a cell will be duplicated at any generation. In these simulations, an offset sigmoid function scaled by a factor of 2 was used for the variable so that its values cross through 0 and asymptotically approach 1: $F\left(x\right)=2\left(\frac{e^{ax}}{e^{ax+1}}\right)-1\;$, where “*x*” equals the number of EBV plasmids per cell and “*a*” is the coefficient for positive selection. This function can be thought of as providing cells with no EBV no advantage so they will not be duplicated, while those with increasing copies of EBV plasmids will be duplicated with a decreasing probability. This selection coefficient was incorporated into the simulation, so that it could be experimentally varied in its magnitude. Testing a range of coefficients yielded an accurate prediction of the distribution of the numbers of EBV plasmids per cell in the dually infected cells (Figure 3A). This prediction required that the coefficient of positive selection be varied along with the number of generations of the infected cells being analyzed (Figure 3B). By setting “a” optimally to 0.1, the distribution at 50 generations was close to the equilibrium value found in cells in culture.

This testing also revealed a link between a selective advantage and the mean number of plasmids per cell: a greater selective advantage leads to a greater average number of plasmids per cell. This link indicates that the selective advantages act dose-dependently. 

### 3.2. KSHV

**Figure 4:**
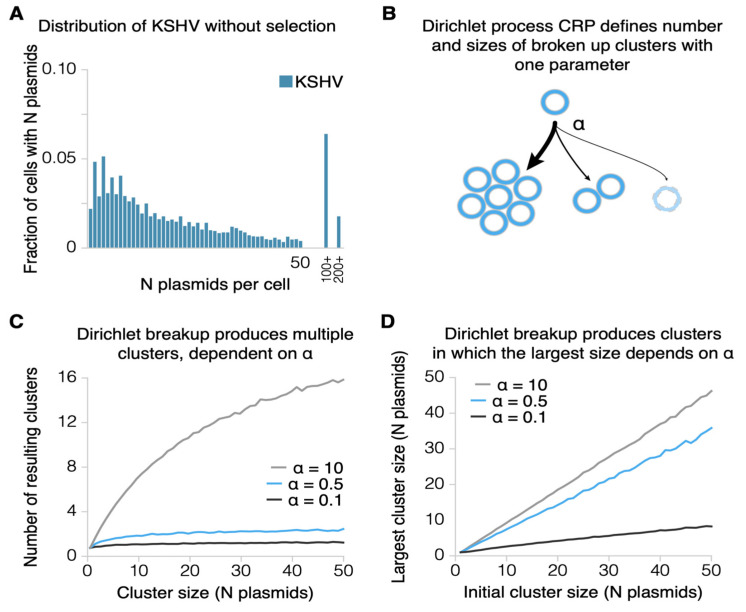
The distribution of KSHV plasmids per cell requires the addition of the modeling of clusters of plasmids and their associated breakup. (**A**) The distribution of KSHV plasmids per cell with no selection. Two bars at the right show the fraction of simulated cells with unrealistically high numbers of plasmids per cell. (**B**) The CRP is entered for each plasmid with a probability of 0.8 (as described in the findings of Chiu et al. [2]). This breaks up clusters, assigning each resulting cluster to the first resulting breakup cluster, and is passed then to successive clusters until a new cluster is formed. This process is represented here with the relative probabilities of forming each size of cluster. (**C**) The CRP produces multiple resulting clusters, the number of which is defined by the “α” parameter. The optimum value of “α” was identified to be 0.5 [2]. (**D**) Varying “α” also affects the resulting cluster sizes, the largest of which are represented here.

Our previous simulations of KSHV’s replication predicted that, over time, the number of molecules per cell in some cells increases to infinity [2]. This continued increase is a result of both the clustering of the viral genomes and the possibility that some daughter cells inherit more and larger clusters. The protein LANA1, encoded by KSHV, binds to both chromatin and histones, linking KHSV both to the chromosome as well as to other molecules of KSHV to form the observed clusters [2,9,10,14]. These clusters lead to a higher average number of plasmids per cell and a lower total population of cells that maintain infection [2]. This distribution can be minimally modeled with three parameters rather than the two used for EBV without selection—a probability of plasmid duplication, a probability of cluster breakup after formation, and a parameter describing the average number of clusters and their sizes after breakup. This third parameter can be fulfilled by the CRP process (a subset of Dirichlet processes termed the Chinese restaurant process). We found in cells infected only with KSHV that KSHV plasmids were duplicated approximately 92% of the time [2]. Using this parameter, a probability of experiencing the CRP of 0.8 per generation, and a CRP *α* parameter of 0.5, yielded the distributions found in vitro in cells infected with only KHSV (Figure 4A–D; additional data in [2]).

### 3.3. Dual Infection

**Figure 5:**
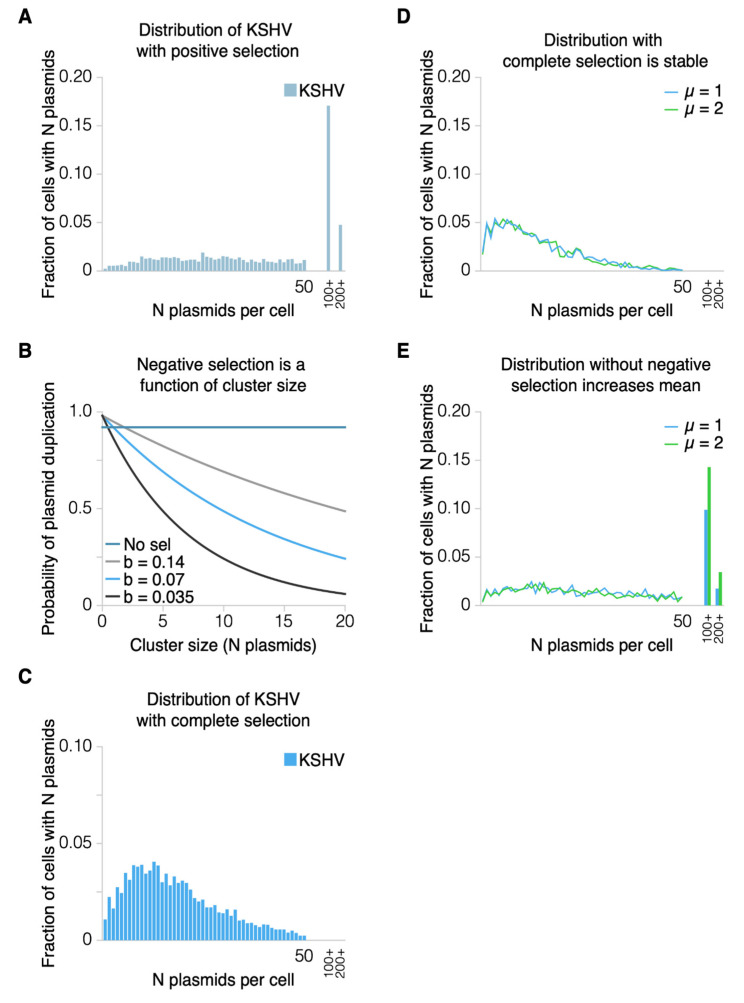
The distributions of KSHV with additional forms of selection are shown. (**A**) A distribution in the same form as Figure 4A, but now calculated with a term for positive selection. The positive selection coefficient and terms are identical to those for EBV, as shown in Figure 3B. Positive selection increases the fraction of cells with unrealistically high numbers of plasmids per cell. (**B**) To model more accurately the results from [16], negative selection was modeled as an exponential decay function describing the probability that any plasmid in a cluster is duplicated as a function of the cluster size. The function was in the form of $F\left(x\right)=de^{-bx}$ in which “*x*” is the size of the cluster; “*d*” is the default duplication probability; and “*b*” is a variable parameter, which we computationally varied and ultimately optimized to be 0.07. (**C**) A distribution in the same form as Figure 4A and Figure 5A, but now calculated with both positive and negative selection. Note that negative selection operating only on clusters eliminates unrealistically high numbers of plasmids per cell. (**D**) In the presence of a positive selective advantage that acts on cells and a negative selective disadvantage that acts on clusters of plasmids, the distribution of plasmids per cell comes to a stable equilibrium regardless of starting conditions. In this case, the blue distribution represents a population that began with each cell containing one plasmid per cell (*μ* = 1). The green distribution began with a population of two plasmids per cell (*μ* = 2). (**E**) Without KSHV paying a penalty for forming clusters, the population does not reach a stable equilibrium. Instead, more cells accumulate ever-increasing numbers of plasmids per cell (*μ* = 1 or 2).

To model the equilibrium found in the average number of KSHV genomes per dually infected cell [16], the same term for a selective advantage used for EBV was included for KSHV. In the absence of a selective advantage, the distribution of KSHV plasmids did not produce an equilibrium, similar to that of EBV. The distribution of KSHV plasmids per cell over the same 50 generations as EBV was calculated, but with an initial m.o.i. of 0.02. This m.o.i. was calculated by measuring the fraction of B-cells infected and corresponded to an m.o.i. of 2–3 when measured on 293 cells [16]. We computed the number of KSHV genomes per cell to approach asymptotically the experimentally determined average number of genomes per cell. Including this term for a selective advantage provided by KSHV did not, however, model our experimental observations; rather, the number of KSHV genomes per cell in some cells still grew to be hundreds or thousands per cell over time (Figure 5A). We have not detected such high numbers of KSHV genomes in dually infected cells [16] and tested two hypotheses to contend with this unrealistic prediction: 1. Can the high numbers of KSHV genomes in a few cells be eliminated by their inhibiting entry of the cells into S-phase (a selective disadvantage that acts on a cell)? 2. Can they be eliminated by their being a disadvantage to the synthesis of KSHV genomes per se (a selective disadvantage that acts on plasmids)? We modeled the first hypothesis by including a selective disadvantage on the probability of replication of a cell proportional to the square of the number of plasmids—a function used in [2] to limit extreme plasmid copy numbers. This produced predictions that did not match experimental data in that the probability of a cell’s replication per generation decreased by 10-fold and, to produce the same mean copy number of plasmids per cell, produced no cells with more than 50 plasmids (Figure S3). In contrast, the second hypothesis produced simulations that reproduced the distributions of KSHV genomes measured per cell in culture [16].

Such a selective disadvantage acting at the level of plasmids is likely to result from the increased barriers for them within a cluster to be replicated. The clustered genomes are bound to each other by LANA1’s carboxy-terminal DNA-binding domain binding site—specifically to the origin sequences within each terminal repeat of KSHV and LANA1’s amino-terminal histone-binding domain binding histones in the nucleosomes wrapped by KSHV’s terminal repeats [2,14,22]. These interactions of LANA1 and KSHV’s origins of DNA synthesis have a parallel in EBV. The binding of EBNA1 to the family of repeats (FR) of OriP in EBV DNA forms a prolonged barrier to fork migration during the S-phase [23]. The findings with EBV lead to the expectation that the multiple interactions of LANA1 with the terminal repeats of KSHV form increasingly stringent barriers to fork migration for the genomes in increasingly large clusters of KSHV genomes. A variable was thus added to the simulation that modified the probability of plasmid duplication as a function of the number of plasmids in the cluster (Figure 1). This variable could be fulfilled by the exponential decay function, $F\left(x\right)=de^{-bx}$, in which “*x*” is the number of plasmids per cluster, “*b*” is the exponential decay coefficient, and “d” is the probability of duplication for plasmids not in clusters (Figure 5B). Genomes in larger clusters each would have diminished rates of being synthesized; the larger the cluster, the less likely it is that its constituents could replicate.

A simulation was developed to measure the probability of plasmid duplication across the population of cells undergoing this mechanism of negative selection. In this case, starting with a mean of 10 plasmids per cell, the simulation was run for 20 generations, and used to give an initial distribution. Another 30 generations were then simulated to determine if the mean probability of plasmid duplication approximated 92%. The simulations were continued for an additional 20 generations and confirmed that they were stable, yielding a duplication probability of 92%. This modelling allowed fitting the probability of duplication for an unclustered plasmid and the different coefficients for the exponential decay function to reproduce a probability of plasmid replication of approximately 92%. The combined modelling yielded a range of pairs of variables (Figure 5B) (for example, a rate of duplication of 92% regardless of cluster size with an exponential decay coefficient of 0, or a rate of duplication of 98% of an unclustered plasmid with an exponential decay coefficient of 0.07). We then tested this range of pairs of parameters on the resulting KSHV distribution and determined that the coefficients of 98% and 0.07 best matched the data (Figure 5C).

**Figure 6:**
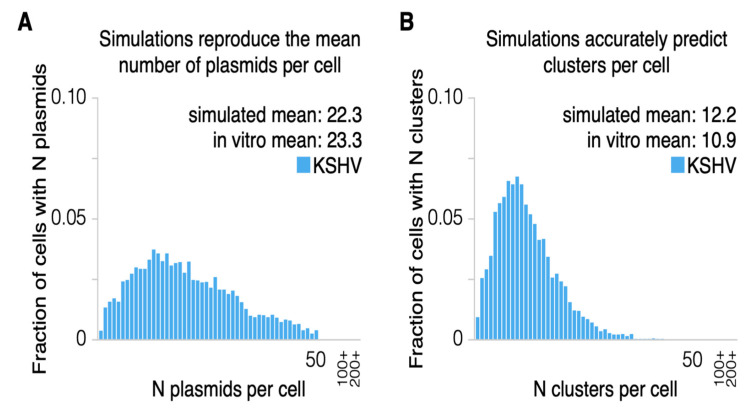
Simulations based on the numbers of KSHV plasmids per cell measured by PCR accurately predict the distribution of clusters per cell measured by FISH [16]. (**A**) Simulations reproduce the mean number of plasmids per cell (blue dot: simulated mean, gray dot: measurements in vitro). As described above, adjusting only the selective advantage coefficient is sufficient to match the mean copy number of plasmids per cell. Here, the coefficient was set optimally to 0.07, which reproduced the mean number of plasmids per cell identified in vitro by qPCR after 20 generations of burn-in. The simulation was run for 30 generations to confirm that the distribution was in equilibrium. (**B**) The mean number of simulated clusters per cell closely matched the counts of plasmids clusters in vitro via FISH [2].

These simulations were developed using conceptually simple forms of selective advantages and disadvantages to achieve an equilibrium in the distributions of EBV and KSHV plasmids in the same cells. Simulating their distributions after 50 generations from starting populations beginning with one plasmid per cell or two plasmids per cell produced indistinguishable distributions with the selective advantages and disadvantages used (Figure 5D). However, in the absence of the selective disadvantage, the population beginning with two plasmids per cell grew to include cells with an unrealistically high number of plasmids per cell (Figure 5E). This finding demonstrates that, for dually infected cells grown in cell culture, there must be a mechanism of selective disadvantage that limits the accumulation of KSHV genomes in them (Figure 1 and Figure 6).

We also tested five of the six in silico identified parameters on data not used previously in our modeling. We simulated a population of dually infected cells described as the “fast population” [16] in stable equilibrium (over 30 generations). We tested whether the simulation accurately represented the number of clusters of plasmids per cell identified by FISH when initialized with the number of plasmids per cell identified by qPCR. This population was selected for fast replication and contained higher copy numbers than other populations. Different populations of cells likely derive different selective advantages from KSHV and EBV. We adjusted the positive selective advantage to 0.07 and found that so doing yielded the measured distributions well (simulated 22.33 plasmids per cell, 12.23 clusters per cell; measured 23.25 ± 5.07 plasmids per cell across five measurements; 10.90 ± 0.38 clusters per cell across two measurements; Figure 6).

## 4. Discussion

What have we learned from our modelling? Our earlier simulations for each viral replicon used limited experimental observations to predict complexities of replication and partitioning of these plasmids successfully [1,2]. For example, modelling of EBV allowed the accurate prediction of the distribution of its plasmids in cells in both the absence and presence of selection [1]. That of KSHV revealed that the clustering of its genomes was necessary for it to be maintained in proliferating cells [2]. Our current modelling began with these earlier simulations with the hypothesis that each replicon behaved independently when in the same host cell. The accuracy of this current modelling validates this hypothesis and is the first insight from this work. It indicates, for example, that the selective advantages provided by both EBV and KSHV to dually infected cells are not mediated by one replicon affecting the synthesis or partitioning of the other.

Our earlier simulations also indicated that both EBV and KSHV would have to provide their host cells selective advantages to be maintained in proliferating cells, but our models dealt only with a binary mechanism of selection (selecting for the presence of at least one plasmid, as in the case of antibiotic resistance [1]). Varying our simulations necessitated incorporating terms for selective advantages that depend on the number of viral genomes per cell. This finding highlights the second insight of this modelling: both EBV and KSHV must benefit their dually infected host cells or be lost from them over time.

A third insight for this modelling illuminates the penalty KSHV pays for clustering its genomes in cells. Our current simulations initially predicted that clusters could grow in numbers of plasmids per cluster without bounds if that growth were neutral for both the virus and the host cell. Our measurements of the number of KSHV genomes per dually infected cell showed that it averages between 10 and 30 molecules per cell [16]. The size of clusters of viral genomes is thus limited and cannot be neutral. We thus tested different hypotheses to learn how to improve the prediction. Our testing led to favoring a mechanism for limiting the size of KSHV clusters based on the finding that EBNA1 bound repetitively to FR comprises a prominent barrier to fork migration during its DNA synthesis [23]. LANA1, because it binds both the many terminal repeats of KSHV directly through its DNA-binding domain and indirectly through its histone-binding domain [14,22], presumably forms more extensive barriers to fork migration the larger the number of replicons per cluster. By including a term for a disadvantage as clusters increase in size, our simulation predicts the observed, stable distribution of KSHV genomes per dually infected cell.

We have modelled the replication and partitioning of EBV and KSHV in peripheral B cells infected in vitro [16]. These infected cells have not been exposed to a host’s immune response and can thus serve as examples of cells when they first encounter that response. They will allow a dissection of how that response affects their evolution towards PELs, a tumor of dually infected B cells that often arises in the background of AIDs, indicating it is favored by a diminished immune response in its host [5].

The framework of these simulations reproduces the complexities of the latent replication of herpesviruses in vitro merely by setting parameters to match the experimental measurements (e.g., the initial distribution of plasmids per cell and the number of generations over which the cells have proliferated) (Figure 1). It can also predict the outcome of experimental manipulations of these replicons. For example, it accurately predicted the behavior of a KSHV plasmid being replicated by a hybrid protein derived from EBV’s EBNA1 and KSHV’s LANA1 [2]. It is thus a powerful experimental complement to genetic dissections of these viral replicons.

## Figures and Tables

**Table 1:** Parameters used in simulations.

In Vitro, Experimentally Derived Parameters	In Silico Derived Parameters	Simulation Parameters
S-phase duplication	Cluster breakup after S-phase	Number of cells to simulate
S-phase equal-partitioning	Cluster CRP alpha	Generations
Presence of clustering	Cluster breakup in S-phase vs. G1 phase	Starting population m.o.i. or starting population mean and standard deviation
Mean of plasmids per cell determined by PCR	Positive selective advantage	
Distribution of plasmids per cell determined by FISH or live-cell imaging in the absence of clustering	Negative selective advantage coefficient	
Distribution of clusters per cell determined by FISH or live-cell imaging		

## Data Availability

The program created to use this model, LatentPlasmidPopulation, is written for Python 3.7+. The source code and extensive documentation can be found at the Github repository found at https://github.com/asugden/plasmid_rep (accessed on 7 July 2021).

## Supplementary Materials

The following are available online at https://www.mdpi.com/article/10.3390/v13081478/s1, Figure S1: A flow diagram of the replication of EBV and KSHV with associated functions. Figure S2: An alternate form of selective advantage does not reproduce a stable equilibrium. S3: An alternate form of selective disadvantage produces unrealistic results. Table S1: Computational parameters and mechanisms for identifying parameter value.
