# Supplementary material for: How Epstein–Barr Virus and Kaposi’s Sarcoma-Associated Herpesvirus Are Maintained Together to Transform the Same B-Cell
Source: Viruses. 2021 Jul 28;13(8):1478. doi: 10.3390/v13081478 (PMC8402831; doi:10.3390/v13081478)
Supplement: Supplementary file 1 [file viruses-13-01478-s001.zip › viruses-1284191-supplementary.pdf]

Supplementary Figure 1

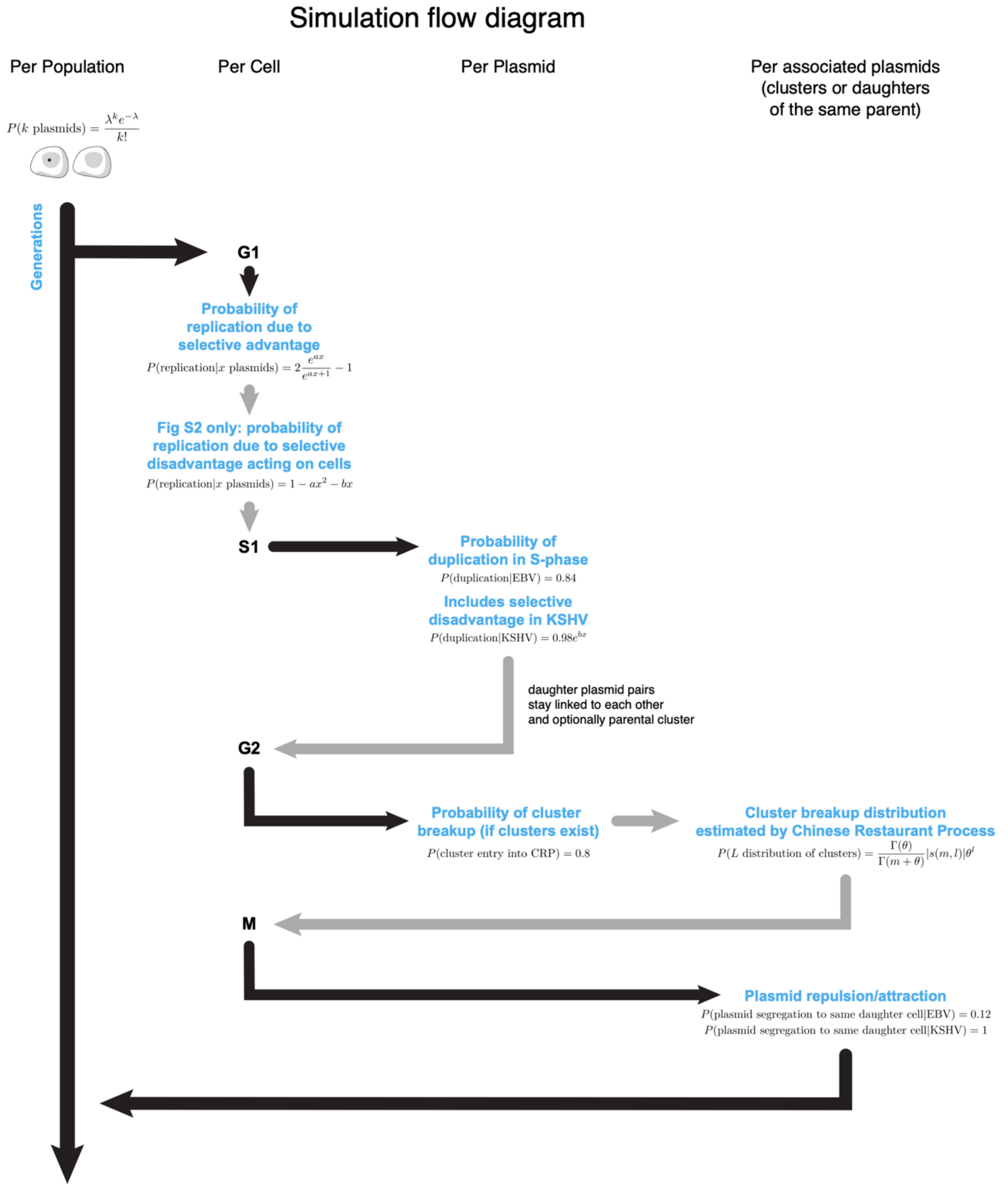

Supplementary Figure S1. A flow diagram of the replication of EBV and KSHV with associated functions. Black lines represent guaranteed paths, dark gray lines represent optional paths. If an optional path is not taken, the simulation returns to the previous column. The four columns are at the population level, the cellular level, the plasmid level, and at the level of associated plasmids.

Supplementary Figure 2

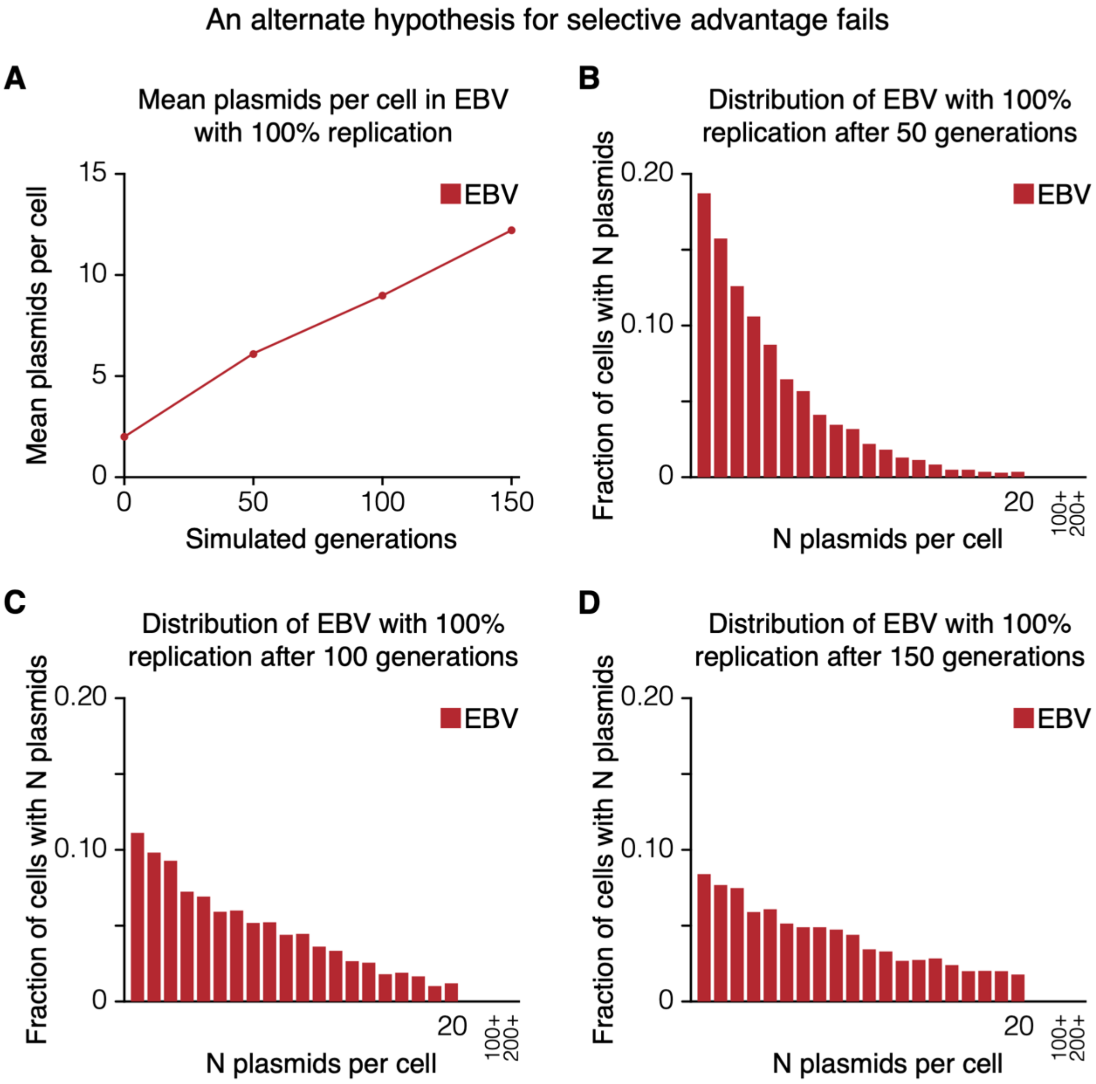

Supplementary Figure S2. An alternate form of selective advantage does not reproduce a stable equilibrium. In particular, 100% faithful replication yields a distribution of plasmids per cell with a mean that increases linearly. (**A**) The mean number of plasmids per cell plotted as a function of simulated generations. Note that a stable equilibrium would appear flat in this diagram. (**B–D**) The distribution of simulated plasmids per cell under 100% replication conditions after 50, 100, and 150 generations, respectively.

Supplementary Figure 3

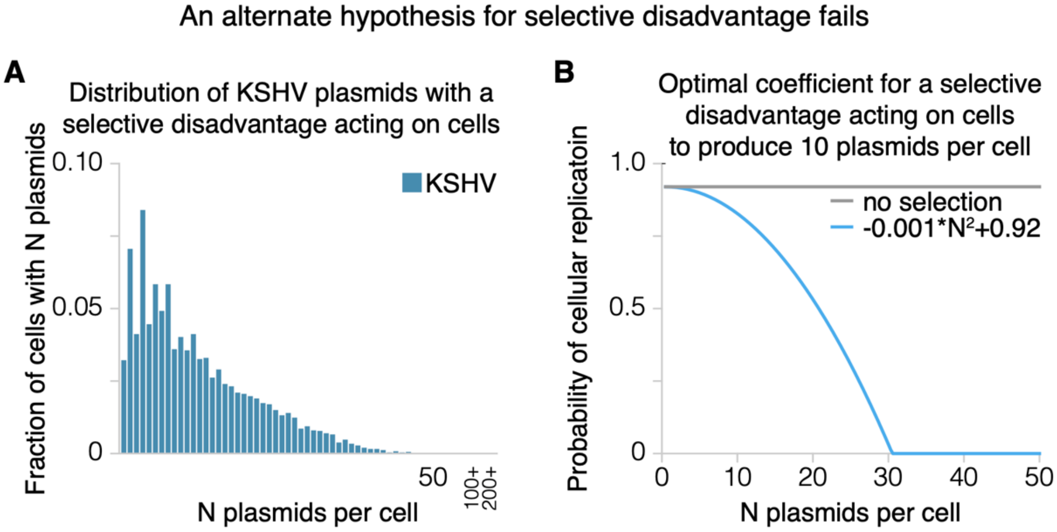

Supplementary Figure S3. An alternate form of selective disadvantage produces unrealistic results. (**A**) A selective disadvantage at the cellular level as a function of numbers of plasmids in the cell yields a truncated distribution. Selective disadvantage was simulated as an increase in generation time with additional plasmids (equivalent to a decreased probability of cell replication per generation). This selective disadvantage this with a quadratic equation as well as a linear equation, not shown, as in our previous work [Chiu et al]. (**B**) To reproduce the same mean number of plasmids per cell, 10, as Figures 3,4, an extreme disadvantage was required that more than doubled the mean generation time of the population and produced zero cells with > 60 plasmids per cell.

Supplementary Table S1.

Computational parameter	Mechanism for identifying parameter value
S-phase duplication	Set from live-cell imaging or PCR without negative selection. Otherwise, computed by tracking the replication probability of every individual plasmid in the simulation across 30 stable generations, followed by confirming with another 20 stable generations
Negative selective advantage	See above
Positive selective advantage	Set by identifying the peak in the distribution of plasmids per cell. Lower values increase the mean number of plasmids per cell.
Cluster breakup	Fit in a large-scale simulation run on a computing cluster varying both this parameter and that of the cluster CRP alpha [2]. Values were matched to distributions of plasmids per cell measured by FISH.
Cluster CRP alpha	See above
Cluster breakup in S-phase vs G1 phase	To model different types of cluster breakup in previous simulations, we allowed cluster breakup to occur in S phase, G1 phase, or both. In this case, the cluster breakup parameter can be distributed across those two phases. Changing this parameter did not produce substantial differences in the final populations of KSHV or EBV and thus is kept constant.
Plasmid-repulsion-attraction	This single parameter represents the probability of segregation to opposite daughter cells 100% of the time at a value of 0 and segregation to the same daughter cell 100% of the time via clustering at a value of 1. We use this single parameter rather than a separate parameter for the presence of clustering and the probability of equal segregation between daughter cells so that we could reproduce a LANA1-EBNA1 fusion protein in [2]. This parameter can fit any state between the extremes of KSHV and EBV.
